# How Global Collaboration Can Improve the Medical Countermeasure Life Cycle for Infectious Disease Outbreaks

**DOI:** 10.1093/infdis/jiae017

**Published:** 2024-01-19

**Authors:** Jessica Swenson, Gary Disbrow, Robert A Johnson

**Affiliations:** Biomedical Advanced Research and Development Authority, Administration for Strategic Preparedness and Response, US Department of Human and Health Services, Washington, District of Columbia, USA; Biomedical Advanced Research and Development Authority, Administration for Strategic Preparedness and Response, US Department of Human and Health Services, Washington, District of Columbia, USA; Biomedical Advanced Research and Development Authority, Administration for Strategic Preparedness and Response, US Department of Human and Health Services, Washington, District of Columbia, USA

**Keywords:** medical countermeasures, emerging infectious disease, international

## Abstract

Infectious disease outbreaks have become increasingly common and require global partnership for adequate preparedness and response. During outbreaks, medical countermeasures (MCMs)—vaccines, therapeutics, and diagnostics—need to reach patients quickly. Recent outbreaks exemplify that products with regulatory approval can expand access and reach patients quicker than investigational products. Unfortunately, insufficient funding globally and differences in funders' prioritization puts gains and future efforts at risk. Of primary concern is (1) lack of a feasible regulatory path and clinical capability to achieve regulatory approval for new MCMs for many diseases; and (2) the need for partners with the mandate, funding, and capabilities to support long-term sustainment of manufacturing capability and stockpiling of licensed products. Without collaboration, the global community runs the risk of losing the capabilities built through years of investment and being underprepared to combat future threats. Synergies between funders are critical to create long-term sustainment of products to ensure access.

## Teaser

Synergies between funders within the medical countermeasure life cycle are critical to ensure long-term sustainment and availability of vaccines, therapeutics, and diagnostics.

Infectious disease outbreaks have become increasingly common and require global partnership for adequate preparedness and response. During outbreaks, medical countermeasures (MCMs)—vaccines, therapeutics, and diagnostics—need to reach patients quickly. The last 20 years have seen great strides in MCMs entering clinical development and, in many cases, achieving regulatory approval, enabling faster, broader access to life-saving products. However, additional collaboration is needed to capitalize on the strengths of all partners involved in outbreak preparedness and response. Partners should leverage global resources, expertise, and stakeholder relationships to ensure MCMs are supported through approval, available when needed, and sustainable for long-term access.

The Biomedical Advanced Research and Development Authority (BARDA) within the Administration for Strategic Preparedness and Response (ASPR) under the US Department of Human and Health Services (HHS), utilizes public-private partnerships to support advanced development of MCMs [1]. The BARDA portfolio supports vaccines, therapeutics, diagnostics, and devices for health security threats, including chemical, biological, radiological, and nuclear health security threats identified by the US Department of Homeland Security through issuance of a Material Threat Determination; pandemic influenza; and emerging infectious diseases. During outbreak response efforts for these threats, BARDA often facilitates access to existing MCMs or supports the rapid development of new, investigational products.

Since 2006, BARDA has catalyzed innovation to protect people during public health emergencies—supporting advanced research and development to enable MCM approval. BARDA is a key player in health security, both in preparedness and response, with strong financial commitment and technical capabilities. BARDA's public-private partnerships have created robust relationships with hundreds of product developers across the globe in vaccines, therapeutics, and diagnostics, leading to 85 United States Food and Drug Administration (US FDA) approvals, licensures, and clearances [2]. BARDA's scientific and regulatory experts frequently collaborate and support domestic and international responses, and our flexible agreements can enable rapid response to public health emergencies within our mission space as funding and science warrants.

Whether enabling access to products already approved with BARDA support, adapting, or rapidly developing new MCMs, our approach is rooted in decades of lessons learned. We support the generation of sound scientific evidence to enable product licensure and subsequent availability, and US FDA approval is the goal for products in our portfolio. During outbreaks, MCMs with data packages that can support approval by stringent regulatory authorities (SRAs), such as the US FDA, European Medicines Agency, and others, can potentially expedite local approvals, SRA collaborative registration, and World Health Organization (WHO) Emergency Use Listing or Prequalification. Regulatory approval is critical because it enables governments and agencies to procure, stockpile, or rapidly manufacture products, supporting prompt deployment during outbreaks.

BARDA has experienced several situations demonstrating how approved MCMs support rapid response and improve access. In 2019, ERVEBO became the world's first US FDA-approved Ebola Zaire vaccine, after multipartner collaboration supported clinical trials, advanced development, and manufacturing [3]. Approval allows this vaccine to be readily available for swift deployment during future outbreaks. In contrast, even with extensive data from investigational vaccines, the coronavirus disease 2019 (COVID-19) vaccine development still took 11 months from start of development until it was available for distribution under US FDA Emergency Use Authorization. However, after authorization of the initial vaccine and identification of correlates of protection, updated vaccine strain formulations to address new COVID-19 variants were able to be developed, manufactured, and authorized quicker, pointing to the speed at which approved products can enable widespread access relative to investigational products [4].

Achieving regulatory approval is a high bar, especially for diseases that occur sporadically and/or in regions with limited clinical trial infrastructure. For pathogens for which alternative regulatory approval pathways, such as US FDA Animal Rule or surrogate markers, are not a feasible option, the challenge is even greater. In these cases, collaboration and innovation in clinical trial design and execution is critical to success. Moving forward, it will take concerted efforts to develop and approve additional MCMs for pathogens with pandemic potential to enable the quickest response and broadest access for outbreaks.

**Figure 1.**
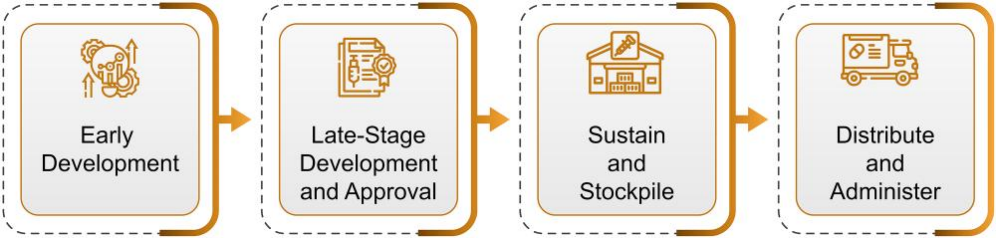
The medical countermeasure life cycle.

Experience also shows that discovery, research and development, and regulatory approval alone are not sufficient to meet US and global preparedness and response goals—access and delivery support is also critical. Comprehensive MCM life cycle management includes not only development, but also stockpiling, sustainment of manufacturing capability, including supply chain and regulatory compliance, and distribution and administration (Figure 1). BARDA's primary role in the MCM life cycle is late-stage development and approval. As part of the later stages of advanced research and development, BARDA also often supports initial stockpiling procurements; however, these are to support US government requirements and are insufficient to address global demand during an outbreak or ensure sustainment.

Within the MCM life cycle, stockpiling and sustaining manufacturing capability is often underappreciated. Sustainment of complex infrastructure, trained staff, and regulatory compliance can often exceed product development costs but are required for long-term, widespread MCM access. Batch manufacturing of products requires firm commitments from purchasers, and low volume or intermittent procurements can be challenging for product sponsors to support.

As an example of this gap, despite significant outreach, there have been minimal to no procurements for approved Ebola therapeutics other than by the US government. The situation is similar for other infectious disease products such as the JYNNEOS vaccine that has been approved for smallpox/mpox since 2019 but has relied largely on the US government supporting procurement for sustainment [5]. The US government procurement alone is likely insufficient to support a sustainable manufacturing capability that could be required for future global poxvirus responses.

The ERVEBO vaccine, in contrast, presents a potential model for how international partnerships can create a more sustainable MCM ecosystem. A collaboration between the product sponsor, BARDA, and Gavi/UNICEF has led to both a national and international stockpile that reduces the burden on individual governments, supports sustainment of manufacturing, and increases access for the countries that need this product most [6]. Additional efforts are required, even for this scenario, but the ERVEBO example demonstrates that partnerships can promote sustainability. Stockpiling and sustainment are responsibilities of the global community and must be adequately prioritized and collectively addressed to respond to future outbreaks efficiently.

While BARDA's mission focus remains on advanced development, approval, and initial stockpiling of MCMs for national preparedness and response, further success depends on solving the issues described above. BARDA's 2022–2026 Strategic Plan calls for engagement with international stakeholders, encouraging alignment and coordination across emerging infectious disease pathogens where BARDA can play a valuable role [7]. As no single agency or funder has the budget and scope needed for global MCM life cycle management for emerging infectious diseases, BARDA is looking to partner with international agencies, regional collaborations, and governments to address these challenges through the following:

1. Supporting global efforts to align on design and strengthen execution of efficacy clinical trials to support regulatory approvals. BARDA appreciates the complexity of this undertaking, the need to have a flexible infrastructure to accommodate the uniqueness of each outbreak, and that many partners will have to work in harmony for this undertaking to be successful. Moreover, with development costs often in the tens of millions of dollars and long lead times, clinical trial material is often in short supply or nonexistent. As part of its support for this overall effort, BARDA plans to further our on-going efforts to facilitate the supply and support of clinical trial material, where possible and as funding permits.
2. In parallel with efficacy trials and other late-stage development efforts, improving coordination among global partners to commit resources toward shared manufacturing needs postapproval, including stockpiling and sustainment. BARDA plans to improve our current efforts to address these challenges in part through facilitating information sharing between manufacturers and funders that can support long-term stockpiling and sustainment of MCMs.

BARDA's unique capabilities, relationships, and experience enable the organization to serve as a critical link within the outbreak response network for threats within our mission space. BARDA will continue to advance the development of critical MCMs toward US FDA approval, supporting regulatory data packages that can expedite local approvals, WHO Emergency Use Listing or Prequalification, and SRA collaborative registration for increased global access. As innovation continues to advance, expanded partnerships can accelerate the development of MCM platforms to support threats across mandates and bolster collective global preparedness.

As BARDA looks to the future, finding partners that complement our mission and support the MCM life cycle will be a key component in deciding which MCM development efforts can be supported. Plans from the global community to support stockpiles and bolster efforts to enable regulatory approval would create an environment where BARDA could consider supporting investments in new MCMs as future priorities emerge. Without collaboration and funding outside of response efforts, the global community runs the risk of losing the capabilities built through years of investment and being underprepared to combat future threats. Synergies between funders that have different roles and responsibilities within the MCM life cycle are critical to MCM availability and create long-term sustainment of products to ensure access.

## Notes

***Acknowledgments***. The authors thank Christopher Houchens, Kimberly Armstrong, Daniel Wolfe, Karen Martins, and Terence Barnhart for their contributions to this perspective.

***Author contributions*.** R. J. and J. S. contributed conceptualization. J. S. prepared the original draft. All authors reviewed and edited the manuscript, and have read and agreed to the published version of the manuscript.

***Financial support*.** No financial support was received for this work.
